# An Evaluation of the Efficacy, Safety, and Tolerability of Abhraloha Compared With Oral Ferrous Ascorbate on Iron Deficiency Anemia in Women: A Randomized Controlled, Parallel-Group, Assessor-Blind Clinical Trial

**DOI:** 10.7759/cureus.14348

**Published:** 2021-04-07

**Authors:** Snehalata Gajbhiye, Paresh G Koli, Maheshkumar Harit, Mrudul Chitrakar, Vishnu Bavane, Mukesh Chawda

**Affiliations:** 1 Pharmacology and Therapeutics, Seth Gordhandas Sunderdas Medical College - King Edward Memorial Hospital, Mumbai, IND; 2 Pharmacology, Seth Gordhandas Sunderdas Medical College - King Edward Memorial Hospital, Mumbai, IND; 3 Pharmacology, Rajiv Gandhi Medical College and Chhatrapati Shivaji Maharaj Hospital, Mumbai, IND; 4 Department of Swasthavritta and Yoga, DY Patil Deemed To Be University School of Ayurveda, Mumbai, IND; 5 Medical Services, Shree Dhootapapeshwar Limited, Mumbai, IND

**Keywords:** iron deficiency anaemia (ida). anaemia of chronic disease(acd), iron profile, iron tablets

## Abstract

Background and objective

Iron deficiency anemia (IDA) is a common condition in women for which ferrous ascorbate (FA) is often prescribed, which can lead to multiple side effects. Abhraloha is an Ayurvedic medicine that has been used for decades in India to treat IDA. In this study, we aimed to evaluate the efficacy and safety of Abhraloha with regard to change in hemoglobin (Hb) levels as compared to the standard treatment using FA in participants with IDA.

Materials and methods

We conducted a single-center, pragmatic, prospective, randomized, active-controlled, two-arm, parallel-group, assessor-blind study to evaluate the efficacy and safety of Abhraloha with regard to change in Hb levels as compared to the standard treatment using FA in participants suffering from IDA. The eligible participants were randomized and were advised to take either Abhraloha (two tablets twice a day) or FA (one tablet twice a day) for eight weeks; they were asked to follow up after 14 days for re-evaluation. On visit 1 and during the study period, the physician assessed the participants on the Pandurog scale and subjective variables. Descriptive statistics were used with unpaired T-test/Mann-Whitney U test for comparison between the groups. The Wilcoxon signed-rank test was used for within-group analysis, and the chi-square test/Fisher’s exact test was employed for categorical data.

Results

Based on our findings, Abhraloha tablets significantly increased all the variables including the Pandurog scale after eight weeks of treatment. Abhraloha reduced total iron-binding capacity (TIBC) and peripheral smear lymphocyte (PSL), which is consistent with an improvement in IDA. There was a statistically significant increase in Hb, red blood cell (RBC) count, packed cell volume (PCV), mean corpuscular volume (MCV), and mean corpuscular hemoglobin (MCH) in the Abhraloha group as compared with the FA group at eight weeks. The Abhraloha group also exhibited a statistically significant improvement in all the subjective variables. Abhraloha was found to be safe and well-tolerated among the participants.

Conclusions

Abhraloha possesses hematinic activity and it improves all the blood indices. It is associated with significantly fewer adverse effects compared to oral iron therapy, which proves that it can be safely used for the treatment of IDA.

## Introduction

Iron deficiency, which is the major cause of anemia, is the most common nutritional disorder worldwide [1]. The National Health Policy 2017 revealed a high level of anemia prevalent among Indian women (53%) [2]. Among the many causes of Iron deficiency anemia (IDA), the most common ones are menstrual loss, increased nutritional demand during pregnancy, early age of childbearing, short intervals between deliveries, repeated pregnancies, and poor access to healthcare [3]. Furthermore, it has been found that consumption of iron is poor among Indian women [4].

The first choice in the treatment of IDA for almost all patients is an oral iron replacement due to its effectiveness, safety, and lower cost [5]. The major problem with oral iron therapy in its classic ferrous form (the most commonly used form) is poor tolerability and a high adverse reaction rate, which is estimated to be as high as 40% in some cases [6]. The most common complaints related to this are nausea, abdominal pain, diarrhea, and constipation. Even though the intravenous form of this therapy has been reported to have better efficacy compared to oral forms [7], it is also associated with various adverse effects of its own. Hence, there is a need to develop newer agents that are both safe and efficacious to treat IDA.

Abhraloha is a time-tested Ayurvedic hematinic formulation. Abhraloha tablets contain natural hematinic ingredients such as Loha Bhasma (processed iron) and Abhraka Bhasma (processed mica). To augment the effects of Loha Bhasma and Abhraka Bhasma, Abhraloha also provides the benefits of the following herbs: Amalaki (Emblica officinalis), Haritaki (Terminalia chebula), Bibhitaka (Terminalia bellirica), Shunthi (Zingiber officinale), Maricha (Piper nigrum), Pippali (Piper longum), Chitraka (Plumbago zeylanica), Musta (Cyperus rotundus), Vidanga (Embelia ribes), and Shatavari (Asparagus racemosus). These herbs are also an integral part of several classical anti-anemic Ayurvedic formulations [8,9]. 

A preclinical study on Abhraloha has reported an increase in hemoglobin (Hb) levels and rectified anemia in iron-deficient animals (unpublished literature). Abhraloha is used in therapeutic practice to treat IDA. However, there is no clinical study to substantiate its efficacy and safety in IDA, and hence we undertook this clinical analysis for the evaluation of Abhraloha in IDA. Our study involved the evaluation of the hematinic activity of Abhraloha in comparison with ferrous ascorbate (FA).

## Materials and methods

The trial was conducted as a single-center, pragmatic, prospective, randomized controlled, assessor-blind study among participants with IDA. The laboratory personnel and the physician were blinded to the allocation of therapy. The study being a pilot study, the sample size was 30 participants in each group. Approval from the Institutional Ethics Committee (IEC) was obtained, and permission and written informed consent were received from each participant before the start of the trial.

After obtaining Clinical Trials Registry - India (CTRI) approval in the first week of February 2019 (CTRI Registration No.: CTRI/2019/01/017303), recruitment was conducted over a period of 8-10 months. Eligible participants were randomly allocated into two groups [Abhraloha group (group A) and FA group (group B)] with a one-to-one allocation ratio and received treatment for eight weeks. In the Abhraloha group, participants were administered two Abhraloha tablets twice a day (four tablets per day) for eight weeks. In the FA group, participants were administered marketed iron preparation providing FA equivalent to elemental iron 100 mg with folic acid 1.5 mg per tablet in a dose of one tablet twice a day (two tablets per day) for eight weeks.

The study was conducted at the Stree Roga and Prasuti Tantra (Obstetrics and Gynecology) OPD, DY Patil Deemed to be University School of Ayurveda, Nerul, Navi Mumbai. Participants were randomly divided into the two treatment groups in the order of their arrival in OPD. Simple randomization was carried out using a computer-generated randomization schedule generated by the random code system for allocation in two groups.

The duration of study participation of each subject was 11 weeks. This period took into account the screening visit, window period, and follow-up visits, including telephonic conversation two weeks (14 days) after the completion of the treatment. The inclusion criteria were as follows: women who were >18 to <65 years old or women in the postpartum period (10 days after delivery), who were diagnosed with IDA with a Hb level of ≤10 g/dL and >7 g/dL and serum ferritin level of <30 µg/L (30 ng/mL), along with a negative urine pregnancy test. The exclusion criteria were as follows: participants having significant vaginal bleeding (estimated blood loss of greater than 100 cc) within the 24 hours prior to randomization, those with a history of anemia other than IDA, those undergoing current treatment with myelosuppressive therapy or asthma therapy, those with bleeding tendency/disorders, with serum transaminases, total protein, and albumin levels of >1.5 the upper limits of normal, serum creatinine level of >1.5 mg/dL, intolerance to iron derivatives, and those with a known history of hepatitis B and/or hepatitis C virus, and a history of HIV or any immunodeficient conditions.

At the baseline visit, investigations were performed to exclude patients based on the exclusion criteria. On visit 1 (day 0), all signs/parameters related to the Pandurog scale and subjective variables were assessed. Participants eligible for the study were randomized according to the randomization sequence and were dewormed with tablet albendazole 400 mg single dose only on visit 1 as a part of deworming protocol, which is a standard of care procedure. Similar steps were followed for visit 2 (day 14 ± 2) and visit 3 (day 28 ± 2) along with complete blood count (CBC) and reticulocyte count for safety assessment. On visit 4 (day 56 ± 2) CBC, liver function tests (LFTs), and renal function tests (RFTs) were done for safety assessment along with physical examination. Participants were telephonically interviewed after two weeks of their last visit to inquire about any adverse events.

The efficacy variables were Hb level, serum ferritin, serum iron, total iron-binding capacity (TIBC), transferrin saturation, red cell indices including mean corpuscular volume (MCV), mean corpuscular hemoglobin (MCH), and mean corpuscular hemoglobin concentration (MCHC), and reticulocyte count. The symptom assessment score used was Pandurog scale (Ayurvedic assessment based on clinical features of Pandurog) with the following grading - grade point 0 (G0): no clinical features/symptoms; grade point 1 (G1): mild clinical features/symptoms; grade point 2 (G2): moderate clinical features/symptoms; and grade point 3 (G3): severe clinical features/symptoms. Adverse events were recorded by the investigator.

Also, subjective variables (symptoms described by the participants and signs recorded by the physician) were recorded from baseline to eight weeks (i.e., after the completion of the treatment). Symptoms described by the participants that were recorded as either Yes or No responses were extreme fatigue, shortness of breath, pale skin, and fast heartbeat. Signs recorded by the physician that were recorded as either Yes or No responses were pallor, dyspnoea, tachycardia, and nail changes (koilonychia).

The primary endpoint was to compare the average change in the Hb level after eight weeks of treatment between the Abhraloha group and the FA group. Secondary endpoints were the changes in average serum ferritin level, average iron profile values, the average red cell indices after eight weeks of treatment between both the groups, average difference in Hb in each of the visits compared to baseline in each of the study arm, average scores in each of the treatment group for each of the clinical features/symptoms of the Pandurog scale from baseline to eight weeks, average scores in each of the treatment group for each of the subjective variable (symptoms described by the participants and signs recorded by the physician) from baseline to eight weeks, and a comparison between the number of participants showing adverse events on clinical and laboratory parameters in two groups.

This was a pilot study, and no formal sample size calculation was done as there are no previous similar studies reported in the literature. The sample size was 30 patients in each group. For statistical analysis, IBM SPSS Statistics Version 25 (IBM, Armonk, NY) was used. The Mann-Whitney U test was used for comparing demographic variables on visit 0 (baseline). The Wilcoxon signed-rank test was used for within-the-group biochemical analysis, and the Mann-Whitney U test was used for between-the-groups biochemical analysis of primary and/or secondary endpoints. Again, the Wilcoxon signed-rank test was used for within-the-group subjective analysis on the Ayurvedic assessment of the Pandurog scale; the Friedman test with the post-hoc test was used for within-the-group analysis of more than two time points, and the Mann-Whitney U test was used for between-the-group analysis. The McNemar’s test was used for within-the-group subjective analysis (described by the participants and recorded by the physician), and for between-the-group analysis, the chi-square test was applied; and if any value in the observation was less than 5, then the Fisher’s exact test was applied. For adverse reaction comparison, the chi-square test was applied, and if any value in the observation was less than 5, then the Fisher’s exact test was applied. A p-value of <0.05 was considered statistically significant.

## Results

**Figure 1:**
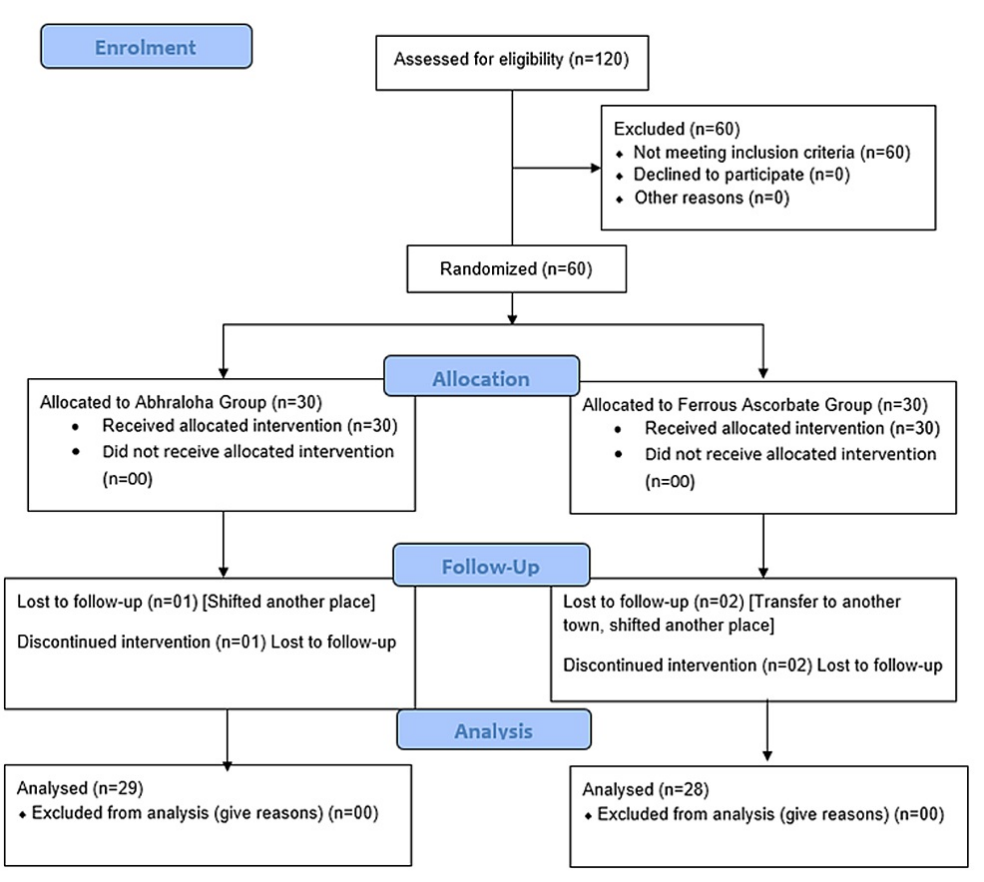
Enrollment flowchart

The sample size of this study was 60, i.e., 30 participants in each group; however, only 57 participants completed the study [n=29 in the Abhraloha group (group A); n=28 in FA group (group B)]. One participant dropped out from the Abhraloha group and did not attend follow-up visit 2. Two participants dropped out from the FA group; one participant did not attend follow-up visit 2, and the other did not attend follow-up visit 3. All the three participants who dropped out did not come for follow-ups since they had moved out of town and did not report for the scheduled follow-up visit (Figure 1).

The mean age of the participants in the Abhraloha group was 36.59 ± 9.19 years and that in the FA group was 35.86 ± 8.13 years. Other baseline variables including Hb, red blood cell (RBC), packed cell volume (PCV), MCV, MCH, MCHC, peripheral smear lymphocyte (PSL), reticulocyte count, serum ferritin, serum iron, TIBC, and transferrin saturation were comparable between the two groups, as shown in Table 1.

The mean Hb level in the Abhraloha group on visit 0 (baseline) was 9.16 ± 1.01 g/dL, which significantly increased to 12.18 ± 0.53 g/dL (p<0.05), as shown in Table 2. A similar finding was observed in the mean Hb level in the FA group. On visit 0, the mean Hb level was 8.99 ± 1.00 g/dL, which significantly increased to 11.50 ± 0.79 g/dL (p<0.05) on visit 4 in the FA group as shown in Table 1. The Hb level in the Abhraloha group on visit 4 was statistically significant when compared with the mean Hb level in the FA group on visit 4 (11.50 ± 0.79 g/dL) (p=0.009) as shown in Table 1.

The mean RBC count in the Abhraloha group on visit 0 (baseline) was 3.64 ± 0.32 million cells/mcL, which significantly increased to 4.39 ± 0.16 million cells/mcL on visit 4 (p<0.05). Similarly, on visit 0, the mean RBC count was 3.65 ± 0.23 million cells/mcL, which significantly increased to 4.23 ± 0.24 million cells/mcL (p<0.05) on visit 4 in the FA group (Table 2). The visit-4 RBC count in the Abhraloha group was statistically significant when compared to that in the FA group: 4.23 ± 0.24 million cells/mcL (p=0.009) (Table 1).

Similarly, for the parameters PCV, MCH, MCHC, serum iron, serum ferritin, and transferrin saturation, a significant increase was observed from visits 0 to 4 in both Abhraloha and FA groups (Table 2). TIBC and PSL count were found to significantly decrease from visits 0 to 4 in both Abhraloha and FA groups (Table 2). For the parameters MCV and reticulocyte count, a significant increase was found from visits 0 to 4 in the Abhraloha group but not in the FA group (Table 2). On visit 4, PCV, MCV (p=0.011), and MCH (p=0.018) levels in the Abhraloha group were statistically significant as compared to those in the FA group (Table 1).

**Table 1:** Biochemical parameters assessed on four visits in the Abhraloha and FA groups *P<0.05 Abhraloha vs. FA group. Between the groups, the Mann-Whitney U test was used SD: standard deviation; FA: ferrous ascorbate; Hb: hemoglobin; RBC: red blood cells; PCV: packed cell volume; MCV: mean corpuscular volume; MCH: mean corpuscular hemoglobin; MCHC: mean corpuscular hemoglobin concentration; TIBC: total iron-binding capacity; PSL: peripheral smear lymphocyte

Parameters	Abhraloha, mean ± SD	Ferrous ascorbate, mean ± SD	P-value (between groups)
Age in years	36.59 ± 9.19	35.86 ± 8.13	0.829
Hb, visit 0 (baseline)	9.16 ± 1.01	8.99 ± 1.00	0.387
Hb, visit 2	9.74 ± 0.92	9.65 ± 0.93	0.648
Hb, visit 3	10.46 ± 0.83	10.34 ± 0.93	0.564
Hb, visit 4	12.18 ± 0.53	11.50 ± 0.79	0.001*
RBC, visit 0 (baseline)	3.64 ± 0.32	3.65 ± 0.23	0.424
RBC, visit 2	3.84 ± 0.30	3.80 ± 0.24	0.285
RBC, visit 3	4.04 ± 0.24	3.99 ± 0.20	0.307
RBC, visit 4	4.39 ± 0.16	4.23 ± 0.24	0.009*
PCV, visit 0 (baseline)	29.78 ± 3.09	29.88 ± 2.78	0.949
PCV, visit 2	31.20 ± 2.79	30.97 ± 2.88	0.513
PCV, visit 3	32.75 ± 2.29	32.20 ± 2.69	0.38
PCV, visit 4	37.27 ± 1.58	35.15 ± 2.33	0.000*
MCV, visit 0 (baseline)	81.67 ± 2.99	81.99 ± 6.07	0.829
MCV, visit 2	81.26 ± 2.13	81.50 ± 7.01	0.949
MCV, visit 3	81.10 ± 1.83	80.73 ± 4.64	0.702
MCV, visit 4	84.92 ± 1.72	83.17 ± 2.93	0.011*
MCH, visit 0 (baseline)	25.09 ± 0.91	24.64 ± 2.09	0.363
MCH, visit 2	25.37 ± 0.67	25.38 ± 2.11	0.867
MCH, visit 3	25.88 ± 0.75	25.93 ± 1.74	0.571
MCH, visit 4	27.75 ± 0.52	27.23 ± 1.17	0.018*
MCHC, visit 0 (baseline)	30.58 ± 0.67	30.33 ± 0.61	0.84
MCHC, visit 2	31.22 ± 0.41	31.16 ± 0.78	0.975
MCHC, visit 3	31.91 ± 0.56	32.11 ± 0.80	0.107
MCHC, visit 4	32.68 ± 0.34	32.73 ± 0.70	0.296
Reticulocyte count, visit 0 (baseline)	1.45 ± 0.17	1.63 ± 0.33	0.63
Reticulocyte count, visit 2	1.50 ± 0.19	1.63 ± 0.28	0.148
Reticulocyte count, visit 3	1.55 ± 0.16	1.62 ± 0.23	0.267
Reticulocyte count, visit 4	1.59 ± 0.15	1.63 ± 0.22	0.497
Serum iron, visit 0 (baseline)	47.00 ± 6.17	43.91 ± 4.80	0.060
Serum iron, visit 4	69.15 ± 6.53	65.74 ± 6.85	0.098
TIBC, visit 0 (baseline)	410.93 ± 24.56	401.24 ± 27.40	0.225
TIBC, visit 4	336.45 ± 25.43	331.27 ± 23.90	0.371
Serum ferritin, visit 0 (baseline)	11.58 ± 3.63	10.99 ± 4.29	0.643
Serum ferritin, visit 4	31.61 ± 5.24	29.06 ± 4.37	0.088
Transferrin saturation, visit 0 (baseline)	11.60 ± 1.95	10.99 ± 1.46	0.078
Transferrin saturation, visit 4	20.69 ± 2.65	19.92 ± 2.35	0.346
PSL, visit 0 (baseline)	31.59 ± 5.51	33.68 ± 5.03	0.20
PSL, visit 2	30.38 ± 4.16	31.39 ± 3.54	0.395
PSL, visit 3	30.86 ± 3.62	31.32 ± 4.00	0.541
PSL, visit 4	29.66 ± 4.15	30.61 ± 3.81	0.526
Grades on Pandurog scale, visit 0	12.83 ± 3.79	14.14 ± 3.31	0.168
Grades on Pandurog scale, visit 4	3.24 ± 3.41	3.57 ± 3.11	0.565

**Table 2:** Biochemical parameters assessed on baseline and final visit in Abhraloha and FA groups ^#^P<0.05 Abhraloha baseline (visit 0) vs. final visit (visit 4); ^$^p<0.05 FA baseline (visit 0) vs. final visit (visit 4). Within the group, the Wilcoxon signed-rank test/Friedman test with the post-hoc test used SD: standard deviation; FA: ferrous ascorbate; Hb: hemoglobin; RBC: red blood cells; PCV: packed cell volume; MCV: mean corpuscular volume; MCH: mean corpuscular hemoglobin; MCHC: mean corpuscular hemoglobin concentration; TIBC: total iron-binding capacity; PSL: peripheral smear lymphocyte

Parameters	Abhraloha, mean ± SD	Ferrous ascorbate, mean ± SD	P-value (Abhraloha within-group vs. visit 0)	P-value (FA within-group vs. visit 0)
Hb, visit 0 (baseline)	9.16 ± 1.01	8.99 ± 1.00		
Hb, visit 4	12.18 ± 0.53	11.50 ± 0.79	0.000^#^	0.000^$^
RBC, visit 0 (baseline)	3.64 ± 0.32	3.65 ± 0.23		
RBC, visit 4	4.39 ± 0.16	4.23 ± 0.24	0.000^#^	0.000^$^
PCV, visit 0 (baseline)	29.78 ± 3.09	29.88 ± 2.78		
PCV, visit 4	37.27 ± 1.58	35.15 ± 2.33	0.000^#^	0.000^$^
MCV, visit 0 (baseline)	81.67 ± 2.99	81.99 ± 6.07		
MCV, visit 4	84.92 ± 1.72	83.17 ± 2.93	0.000^#^	0.000^$^
MCH, visit 0 (baseline)	25.09 ± 0.91	24.64 ± 2.09		
MCH, visit 4	27.75 ± 0.52	27.23 ± 1.17	0.000^#^	0.000^$^
MCHC, visit 0 (baseline)	30.58 ± 0.67	30.33 ± 0.61		
MCHC, visit 4	32.68 ± 0.34	32.73 ± 0.70	0.000^#^	0.000^$^
Reticulocyte count, visit 0 (baseline)	1.45 ± 0.17	1.63 ± 0.33		
Reticulocyte count, visit 4	1.59 ± 0.15	1.63 ± 0.22	0.000^#^	0.000^$^
Serum iron, visit 0 (baseline)	47.00 ± 6.17	43.91 ± 4.80		
Serum iron, visit 4	69.15 ± 6.53	65.74 ± 6.85	0.000^#^	0.000^$^
TIBC, visit 0 (baseline)	410.93 ± 24.56	401.24 ± 27.40		
TIBC, visit 4	336.45 ± 25.43	331.27 ± 23.90	0.000^#^	0.000^$^
Serum ferritin, visit 0 (baseline)	11.58 ± 3.63	10.99 ± 4.29		
Serum ferritin, visit 4	31.61 ± 5.24	29.06 ± 4.37	0.000^#^	0.000^$^
Transferrin saturation, visit 0 (baseline)	11.60 ± 1.95	10.99 ± 1.46		
Transferrin saturation, visit 4	20.69 ± 2.65	19.92 ± 2.35	0.000^#^	0.000^$^
PSL, visit 0 (baseline)	31.59 ± 5.51	33.68 ± 5.03		
PSL, visit 4	29.66 ± 4.15	30.61 ± 3.81	0.026^#^	0.001^$^
Grades on Pandurog scale, visit 0	12.83 ± 3.79	14.14 ± 3.31		
Grades on Pandurog scale, visit 4	3.24 ± 3.41	3.57 ± 3.11	0.000^#^	0.000^$^

The mean of assessment grades on the Pandurog scale (Ayurvedic assessment based on clinical features of Pandurog) in the Abhraloha group on visit 1 was 12.83 ± 3.79, which significantly decreased to 3.24 ± 3.41 on visit 4 (p<0.05) (Table 2). A similar finding was observed in the mean Ayurvedic assessment grade in the FA group. On visit 1, the mean Ayurvedic assessment grade was 14.14 ± 3.31, which significantly decreased to 3.57 ± 3.11 (p<0.05) on visit 4 in the FA group (Table 2). However, the mean Ayurvedic assessment grade in the Abhraloha group on visit 4 (3.24 ± 3.41) was not statistically significant when compared to the same in the FA group (3.57 ± 3.11) (p=0.564) (Table 2).

All subjective variables assessed on the Ayurvedic-based Pandurog scale in the Abhraloha group showed a statistically significant reduction from visit 1 to visit 4, except for the symptom of Shotha (edema) (p=0.317), which did not show a significant reduction. Similar findings were noted in the FA group where all the parameters assessed on the Ayurvedic-based Pandurog scale showed a statistically significant reduction from visit 1 to visit 4, except for the symptom of Shotha (Edema) (p=0.083). The visit-4 grades for individual parameters in the Abhraloha group were not statistically significant when compared to the same in the FA group (Table 3).

**Table 3:** Subjective variables assessed on Ayurvedic-based Pandurog scale in Abhraloha and FA groups ^#^P<0.05 Abhraloha baseline (visit 0) vs. final visit (visit 4); *p<0.05 FA baseline (visit 0) vs. final visit (visit 4); ^$^p<0.05 Abhraloha vs. FA. Within the group, the Wilcoxon signed-rank test/Friedman test with the post-hoc test used SD: standard deviation; FA: ferrous ascorbate

Variables	Abhraloha, mean ± SD	Ferrous ascorbate, mean ± SD	P-value (Abhraloha vs. FA)	P-value (Abhraloha within-group vs. visit 0)	P-value (FA within-group vs. visit 0)
Vaivarnata (pallor), visit 1	2.17 ± 0.66	2.25 ± 0.75	0.542		
Vaivarnata (pallor), visit 2	2.10 ± 0.67	2.14 ± 0.71	0.779		
Vaivarnata (pallor), visit 3	1.66 ± 0.9	1.79 ± 0.83	0.640		
Vaivarnata (pallor), visit 4	0.44 ± 0.75	0.71 ± 0.81	0.161	0.000^#^	0.000*
Daurbalya (weakness), visit 1	2.62 ± 0.49	2.75 ± 0.44	0.298		
Daurbalya (weakness), visit 2	2.45 ± 0.57	2.19 ± 0.62	0.112		
Daurbalya (weakness), visit 3	1.79 ± 0.68	1.75 ± 0.70	0.929		
Daurbalya (weakness), visit 4	0.93 ± 0.78	0.86 ± 0.71	0.758	0.000^#^	0.000*
Shrama (fatigue), visit 1	2.28 ± 0.53	2.68 ± 0.48	0.005		
Shrama (fatigue), visit 2	1.79 ± 0.68	2.00 ± 0.61	0.264		
Shrama (fatigue), visit 3	1.28 ± 0.65	1.54 ± 0.64	0.224		
Shrama (fatigue), visit 4	0.59 ± 0.69	0.79 ± 0.79	0.369	0.000^#^	0.000*
Aruchi (anorexia), visit 1	1.86 ± 0.88	1.96 ± 0.74	0.770		
Aruchi (anorexia), visit 2	1.24 ± 0.74	1.61 ± 0.88	0.089		
Aruchi (anorexia), visit 3	0.83 ± 0.76	1.14 ± 0.80	0.133		
Aruchi (anorexia), visit 4	0.30 ± 0.61	0.46 ± 0.69	0.291	0.000^#^	0.000*
Adhirata (irritability), visit 1	2.21 ± 0.73	2.39 ± 0.57	0.367		
Adhirata (irritability), visit 2	1.76 ± 0.79	1.89 ± 0.57	0.453		
Adhirata (irritability), visit 3	1.45 ± 0.78	1.39 ± 0.88	0.836		
Adhirata (irritability), visit 4	0.70 ± 0.82	0.57 ± 0.69	0.629	0.000^#^	0.000*
Shwasa (dyspnoea), visit 1	1.21 ± 0.98	1.36 ± 0.99	0.574		
Shwasa (dyspnoea), visit 2	0.66 ± 0.77	1.18 ± 0.98	0.040^$^		
Shwasa (dyspnoea), visit 3	0.41 ± 0.73	0.64 ± 0.68	0.110		
Shwasa (dyspnoea), visit 4	0.15 ± 0.46	0.18 ± 0.39	0.527	0.000^#^	0.000*
Hridayspandan (palpitation), visit 1	0.31 ± 0.60	0.54 ± 0.74	0.208		
Hridayspandan (palpitation), visit 2	0.21 ± 0.41	0.29 ± 0.60	0.839		
Hridayspandan (palpitation), visit 3	0.03 ± 0.19	0.21 ± 0.50	0.077		
Hridayspandan (palpitation), visit 4	0 ± 0	0 ± 0	1.000	0.023^#^	0.002*
Shotha (edema), visit 1	0.10 ± 0.41	0.11 ± 0.31	0.649		
Shotha (edema), visit 2	0.10 ± 0.41	0.11 ± 0.31	0.649		
Shotha (edema), visit 3	0.03 ± 0.19	0.07 ± 0.26	0.536		
Shotha (edema), visit 4	0 ± 0	0 ± 0	1.000	0.317	0.083
Total, visit 1	12.83 ± 3.79	14.14 ± 3.31	0.168		
Total, visit 2	10.38 ± 3.69	11.46 ± 3.39	0.242		
Total, visit 3	7.48 ± 3.70	8.61 ± 3.36	0.210		
Total, visit 4	3.24 ± 3.41	3.57 ± 3.11	0.565	0.000^#^	0.000*

In the subjective variables, i.e., symptoms described by the participants, a statistically significant (p<0.05) improvement was seen in extreme fatigue, shortness of breath, and pale skin on visit 4 when compared to visit 1 in the Abhraloha group. However, no statistically significant (p>0.05) improvement was seen in terms of the fast heartbeat on visit 4 as compared to visit 1 (Table 4). Similar findings were observed in the FA group in which all symptoms showed statistically significant (p<0.05) improvement on visit 4 when compared to visit 1 (Table 4). However, no significant difference was seen between Abhraloha and FA groups.

In the subjective variables, i.e., signs recorded by the physician, a statistically significant (p<0.05) improvement was seen in pallor, dyspnoea, and tachycardia on visit 4 when compared to visit 1 in the Abhraloha group. Similar findings were observed in the FA group where only pallor and dyspnoea showed statistically significant (p<0.05) improvement on visit 4 when compared to visit 1 (Table 4). However, no significant difference was seen between Abhraloha and FA groups. Both Abhraloha and FA groups did not show any significant difference in nail changes from visit 1 to visit 4 (Table 5).

**Table 4:** Assessment of subjective variables (symptoms described by the participants) in Abhraloha and FA groups ^#^P<0.05 Abhraloha visit 1 vs. final visit (visit 4); *p<0.05 FA baseline visit 1 vs. final visit (visit 4), using chi-square test FA: ferrous ascorbate

Variables	Abhraloha (n=29)	Ferrous ascorbate (n=28)	P-value for Abhraloha (visit 4 vs. visit 1)	P-value for FA (visit 4 vs. visit 1)
Extreme fatigue, visit 1	15	16		
Extreme fatigue, visit 2	14	15		
Extreme fatigue, visit 3	6	6		
Extreme fatigue, visit 4	1	2	<0.05^#^	<0.05*
Shortness of breath, visit 1	16	16		
Shortness of breath, visit 2	10	9		
Shortness of breath, visit 3	3	2		
Shortness of breath, visit 4	1	0	<0.05^#^	<0.05*
Pale skin, visit 1	27	27		
Pale skin, visit 2	24	24		
Pale skin, visit 3	15	16		
Pale skin, visit 4	6	7	<0.05^#^	<0.05*
Fast heartbeat, visit 1	8	10		
Fast heartbeat, visit 2	4	7		
Fast heartbeat, visit 3	2	3		
Fast heartbeat, visit 4	3	0	>0.05	<0.05*

**Table 5:** Assessment of subjective variables (signs recorded by the physician) in Abhraloha and FA groups ^#^P<0.05 Abhraloha visit 1 vs. final visit (visit 4); *p<0.05 FA baseline visit 1 vs. final visit (visit 4), using chi-square test FA: ferrous ascorbate

Variables	Abhraloha (n=29)	Ferrous ascorbate (n=28)	P-value for Abhraloha (visit 4 vs. visit 1)	P-value for FA (visit 4 vs. visit 1)
Pallor, visit 1	23	27		
Pallor, visit 2	20	20		
Pallor, visit 3	16	16		
Pallor, visit 4	5	7	>0.05	>0.05
Dyspnoea, visit 1	14	16		
Dyspnoea, visit 2	10	11		
Dyspnoea, visit 3	4	2		
Dyspnoea, visit 4	1	1	>0.05	>0.05
Tachycardia, visit 1	9	4		
Tachycardia, visit 2	2	3		
Tachycardia, visit 3	1	1		
Tachycardia, visit 4	0	0	<0.05^#^	<0.05*
Nail changes, visit 1	2	2		
Nail changes, visit 2	2	2		
Nail changes, visit 3	1	1		
Nail changes, visit 4	1	1	>0.05	>0.05

Table 6 summarizes the adverse drug reactions in both groups. One participant in the FA group and three participants in the Abhraloha group had a fever on visit 2. One participant in the FA group had a cough on visit 3. One participant had a weakness on visit 3 in the Abhraloha Group. In all the participants of both Abhraloha and FA groups, the RFTs [serum creatinine and blood urea nitrogen (BUN)] and LFTs [serum glutamic-oxaloacetic transaminase/aspartate aminotransferase (SGOT/AST) and serum glutamic-pyruvic transaminase/alanine aminotransferase (SGPT/ALT)] on visit 0 (baseline) and visit 4 were in the acceptable normal range. Compliance/adherence in taking study medications was well within the defined normal range among participants of both groups. None of the participants from both groups reported any adverse events two weeks after their last visit.

**Table 6:** Adverse drug reactions reported in Abhraloha and FA groups FA: ferrous ascorbate

Adverse reactions	Abhraloha (n=29)	Ferrous ascorbate (n=28)
Diarrhea, visit 0 (baseline)	0	0
Diarrhea, visit 2	0	0
Diarrhea, visit 3	0	0
Diarrhea, visit 4	0	0
Constipation, visit 0 (baseline)	1	0
Constipation, visit 2	1	2
Constipation, visit 3	1	5
Constipation, visit 4	0	5
Nausea or vomiting, visit 0	1	0
Nausea or vomiting, visit 2	0	1
Nausea or vomiting, visit 3	0	0
Nausea or vomiting, visit 4	0	0
Heartburn, visit 0 (baseline)	2	0
Heartburn, visit 2	0	3
Heartburn, visit 3	0	0
Heartburn, visit 4	0	0
Leg pain, visit 0 (baseline)	2	1
Leg pain, visit 2	1	4
Leg pain, visit 3	3	1
Leg pain, visit 4	0	0
Darkened skin/urine color, visit 0 (baseline)	0	0
Darkened skin/urine color, visit 2	0	0
Darkened skin/urine color, visit 3	1	0
Darkened skin/urine color, visit 4	0	0
Sore throat, visit 0 (baseline)	0	0
Sore throat, visit 2	1	0
Sore throat, visit 3	0	0
Sore throat, visit 4	0	0
Trouble swallowing, visit 0 (baseline)	0	0
Trouble swallowing, visit 2	1	0
Trouble swallowing, visit 3	2	0
Trouble swallowing, visit 4	0	0
Severe stomach pain, visit 0 (baseline)	0	0
Severe stomach pain, visit 2	0	1
Severe stomach pain, visit 3	1	0
Severe stomach pain, visit 4	0	0
Blood in stools, visit 0 (baseline)	0	0
Blood in stools, visit 2	1	0
Blood in stools, visit 3	0	1
Blood in stools, visit 4	0	0
Fever, visit 0 (baseline)	1	0
Fever, visit 2	3	1
Fever, visit 3	1	0
Fever, visit 4	0	0
Other adverse events	Weakness (1 participant)	Cough (1 participant)

## Discussion

In our study, we found that there was an increase in Hb levels in both the Abhraloha and FA groups when compared to each other and with the baseline values. Oral iron is the gold standard to treat mild-to-moderate IDA [10]. The major problem with oral iron therapy in its classic ferrous form is poor tolerability and its high adverse reaction rate, which can be as high as 40% in some cases [6]. The most common complaints are nausea, abdominal pain, diarrhea, and constipation. Even though it has been reported that the intravenous form has better efficacy in comparison with oral forms [7], it is also associated with certain adverse effects. Hence, there is an urgent need to develop newer agents that are both safe and efficacious to treat IDA.

Ayurveda can provide effective and safe or tolerable iron supplements, and therefore the present study was planned to evaluate the efficacy and safety of an Ayurvedic herbomineral formulation, Abhraloha, in the treatment of IDA. In our study, we observed that Abhraloha significantly increased all the variables such as Hb, RBC count, PCV, MCV, MCH, MCHC, reticulocyte count, serum ferritin, serum iron, and transferrin saturation after eight weeks of treatment. Abhraloha reduced TIBC and PSL, which was consistent with an improvement in IDA.

The increase in Hb, RBC count, PCV, MCV, and MCH in the Abhraloha group was statistically significant when compared with the FA group after eight weeks of treatment. These results show that Abhraloha may help increase these variables in a short period of eight weeks as compared to FA. Increased levels of TIBC suggest that total iron body stores are low, and thus the iron-binding sites on transferrin increase [11]. In this study, the TIBC reduced after treatment with iron supplementation and Abhraloha therapy.

IDA is characteristically identified as microcytic (low MCH and MCHC), hypochromic anemia (low MCV) [12]. Our study also found that MCV, MCH, and MCHC were decreased in participants, which is a sign of IDA, and improvement in these parameters reinforces the efficacy of iron supplementation and Abhraloha. The low reticulocyte count is a sign of IDA [13]. Thus, the finding in our study is consistent with that of Butthep et al., and the reticulocyte count improved after treatment with FA and Abhraloha.

Iron studies diagnostic for IDA anemia reveal a low serum iron (<7.1 µg/L) [14]. Another study has shown that Hb production had a relationship with the level of serum iron. In IDA with oral iron supplementation, patients could achieve serum iron values between 70 µg/100 ml and 150 µg/100 ml, and RBC production increased to four to five times the normal levels. With intravenous iron dextran, the iron supply increased the serum iron to values greater than 200 µg/100 ml with a concomitant increase in marrow production to 4.5-7.8 times the normal levels [15]. In our study, the serum iron levels were low at baseline, and they improved significantly after the therapy with the study drugs, which is consistent with the findings of Johnson-Wimbley and Graham [15].

Iron plays an essential role in the surveillance of immune cells and is necessary for monocyte/macrophage differentiation [16,17]. In a study by Aly et al., IDA patients recorded a higher lymphocyte count as compared to the controls [18]. In our study, we recorded a higher PSL percentage, which reduced after treatment with FA and Abhraloha.

The objective improvement with Abhraloha treatment was also visible from the improvement in subjective variables. Subjective assessment based on the Pandurog scale showed statistically significant improvement in grades of symptoms in both the Abhraloha and FA groups. Each symptom on the Pandurog scale showed a significant reduction in both Abhraloha and FA groups from visit 1 to visit 4 (i.e., after the completion of eight weeks treatment), except in the symptom of Shotha (edema). This could be because the baseline score for this parameter was between 0-1, indicating that patients had mild or no symptoms at baseline itself. Also, the study was for a short duration of eight weeks, and therefore the improvement that could have occurred after that time frame was not seen by us.

The subjective variables (symptoms described by the participants) like extreme fatigue, shortness of breath, and pale skin showed statistically significant improvement after eight weeks of treatment in the Abhraloha group, except for the fast heartbeat, which did not reveal any significant improvement. The FA group also exhibited statistical improvement in all the variables.

The subjective variables (signs recorded by the physician) like pallor, dyspnoea, and tachycardia showed statistically significant improvement after eight weeks of treatment with Abhraloha. However, the FA group showed statistically significant improvement only in pallor and dyspnoea. Both Abhraloha and FA groups did not show any improvement in nail changes after eight weeks of treatment. The inability of nail changes to revert could be because of the long duration (approximately four months of treatment) that is required for the same to occur [19].

The adverse effects were similar in both groups except for constipation, which was significantly higher in the FA group. This finding is also consistent with FA and other oral iron preparations having constipation as a common adverse effect [20].

IDA is correlated with Pandurog, which is described as a Rasa Pradoshaja Vikara, wherein Alpa Rakta (decreased blood) is one of the key features along with Panduta (paleness) [21]. Abhraloha is a Rasayana formulation that supports the function of Agni (metabolism) to produce quality Aahara Rasa (nutrition) that subsequently results in enhanced formation of Rakta Dhatu (blood tissue) while maintaining the Dosha balance (functional balance of Tri-Dosha elements) in the body.

Haritaki (Terminalia chebula), Bibhitaka (Terminalia bellirica), and Amalaki (Emblica officinalis) in combination are known as Triphala and are well known for their gastroprotective, immunomodulatory, and rejuvenating actions [22]. The combination of Shunthi (Zingiber officinale), Maricha (Piper nigrum), and Pippali (Piper longum) is called Trikatu and is well known to improve appetite and digestion. It is also considered a bioavailability enhancer [23,24]. The combination of Chitraka (Plumbago zeylanica), Musta (Cyperus rotundus), and Vidanga (Embelia ribes) is referred to as Trimada. Besides its appetizing and digestive actions, it is also associated with anthelmintic action [25]. Shatavari (Asparagus racemosus) is a versatile feminine tonic and is well known for its gastroprotective, anti-hepatotoxic, immunomodulatory, and galactagogue actions [26]. In an experimental model of anemia in rats, it has been reported to possess anti-anemic activity [27].

The positive role of Abhraloha in the treatment of IDA documented in this clinical study is consistent with a previously conducted experimental study (unpublished data) in an animal model of IDA in Wistar rats. In this study, Abhraloha was administered daily by oral route for 21 days. The results showed that Abhraloha treatment in IDA in the preclinical model produced an equivalent increase in Hb, RBC, and serum iron levels and decreased serum iron-binding capacity, which was similar to the results with ferrous sulfate treatment.

The prime hematinic in Abhraloha is Loha Bhasma, which is well-known for its hematinic activity. An in-vivo study was carried out to evaluate the hematinic effect of two well-known and commonly used Ayurvedic iron preparations of iron: Lauha Bhasma and Mandura Bhasma. This study was conducted in Charles Foster strain rats by inducing anemia by the administration of mercuric chloride (9 mg/kg). The results suggested that Lauha Bhasma and Mandura Bhasma (11 mg/kg) possess significant (p<0.05) hematinic and cytoprotective activity [28]. Also, an in-vivo study on Lauha Bhasma as a potential hematinic agent in anemia states that Lauha Bhasma could reverse anemia induced by phenylhydrazine in Wistar rats. Thus, it was concluded that Lauha Bhasma is a very effective Ayurvedic hematinic and can be useful in the treatment of anemia [29]. After reviewing results of this study and preclinical studies on Abhraloha and hematinics like Lauha Bhasma and Mandura Bhasma, it is clear that Abhraloha has hematinic quality as it improves all the blood indices and is associated with a lower incidence of adverse effects compared to oral iron therapy, which proves that it can safely treat IDA. More clinical studies and clinical trials should be conducted among larger populations to strengthen and validate the results of our study. The major limitation of this study is that it was not blinded. However, since the study had objective variables as primary outcome parameters, the lack of blinding could not have affected the results.

## Conclusions

Based on our findings, Abhraloha possesses hematinic activity as it improves all the blood indices and is associated with a lower incidence rate of adverse effects compared to oral iron therapy, which proves that it can be safely used to treat IDA.

## References

[1] Chaparro CM, Suchdev PS (2019). Anemia epidemiology, pathophysiology, and etiology in low- and middle-income countries. Ann N Y Acad Sci.

[2] NATIONAL HEALTH POLICY, 2017 2017 (2020). Government of India: Ministry of Health & Family Welfare - National Health Policy 2017. https://www.nhp.gov.in/nhpfiles/national_health_policy_2017.pdf.

[3] Scholl TO (2011). Maternal iron status: relation to fetal growth, length of gestation, and iron endowment of the neonate. Nutr Rev.

[4] Rai RK, Fawzi WW, Barik A, Chowdhury A (2018). The burden of iron-deficiency anaemia among women in India: how have iron and folic acid interventions fared?. WHO South East Asia J Public Health.

[5] Cançado RD, de Figueiredo PO, Olivato MC, Chiattone CS (2011). Efficacy and safety of intravenous iron sucrose in treating adults with iron deficiency anemia. Rev Bras Hematol Hemoter.

[6] Bhavi SB, Jaju PB (2017). Intravenous iron sucrose v/s oral ferrous fumarate for treatment of anemia in pregnancy. A randomized controlled trial. BMC Pregnancy Childbirth.

[7] Van Wyck DB, Roppolo M, Martinez CO, Mazey RM, McMurray S (2005). A randomized, controlled trial comparing IV iron sucrose to oral iron in anemic patients with nondialysis-dependent CKD. Kidney Int.

[8] Samal J (2016). Ayurvedic preparations for the management of iron deficiency anemia: a systematic review. Ayu.

[9] Daya Shankar NHM (2014). Clinical evaluation of an ayurvedic preparation or the treatment of iron deficiency anemia in patients. J Homeopath Ayurvedic Med.

[10] Breymann C, Dudenhausen JW (2017). Iron deficiency in women. Handbook of Famine, Starvation, and Nutrient Deprivation.

[11] Porter JB, Garbowski MW (2018). Interaction of transfusion and iron chelation in thalassemias. Hematol Oncol Clin North Am.

[12] Sarma PR (1990). Red cell indices. Clinical Methods: The History, Physical, and Laboratory Examinations. 3rd edition.

[13] Butthep P, Wisedpanichkij R, Jindadamrongwech S, Kaewkethong P, Pattamakom S, Sila-Asna M, Bunyaratvej A (2000). Reticulocyte analysis in iron deficiency anemia and hemolytic anemia. J Med Assoc Thai.

[14] Bermejo F, García-López S (2009). A guide to diagnosis of iron deficiency and iron deficiency anemia in digestive diseases. World J Gastroenterol.

[15] Johnson-Wimbley TD, Graham DY (2011). Diagnosis and management of iron deficiency anemia in the 21st century. Therap Adv Gastroenterol.

[16] Kosch M, Schaefer RM (2002). Indication and practical implementation of parenteral iron therapy. Wien Klin Wochenschr.

[17] Collins HL (2003). The role of iron in infections with intracellular bacteria. Immunol Lett.

[18] Aly SS, Fayed HM, Ismail AM, Abdel Hakeem GL (2018). Assessment of peripheral blood lymphocyte subsets in children with iron deficiency anemia. BMC Pediatr.

[19] Razmi T M, Nampoothiri RV, Dogra S (2018). Koilonychia in iron deficiency. QJM.

[20] Panchal PJ, Desai MK, Shah SP, Solanki MN (2015). Evaluation of efficacy, safety and cost of oral and parenteral iron preparations in patients with iron deficiency anemia. J Appl Pharm Sci.

[21] Rai S, Kar AC (2016). A review on role of psychological factors in the etiopathogenesis of Pandu Roga with reference to iron deficiency anemia. Ayu.

[22] Peterson CT, Denniston K, Chopra D (2017). Therapeutic uses of Triphala in ayurvedic medicine. J Altern Complement Med.

[23] Atal N, Bedi KL (2010). Bioenhancers: revolutionary concept to market. J Ayurveda Integr Med.

[24] Kaushik R, Jain J, Khan AD, Rai PD (2018). Trikatu - a combination of three bioavailability enhancers. Int J Green Pharm.

[25] Salunke M, Bhalerao S (2016). Anti-obesity effect of Trimad, a polyherbal formulation: a review. Int J Pharm Sci Rev Res.

[26] Alok S, Jain SK, Verma A, Kumar M, Mahor A, Sabharwal M (2013). Plant profile, phytochemistry and pharmacology of Asparagus racemosus (Shatavari): a review. Asian Pacific J Trop Dis.

[27] Miller JL (2013). Iron deficiency anemia: a common and curable disease. Cold Spring Harb Perspect Med.

[28] Sarkar PK, Prajapati PK, Choudhary AK, Shukla VJ, Ravishankar B (2007). Haematinic evaluation of Lauha Bhasma and Mandura Bhasma on HgCl 2-induced anemia in rats. Indian J Pharm Sci.

[29] Potbhare MS, Tenpe C, Deepak S (2016). Evaluation of Ayurvedic preparation lauha bhasma as a potential haematinic agent. Int J Pharmacol Res.

